# Production of Laccase by* Cochliobolus *sp. Isolated from Plastic Dumped Soils and Their Ability to Degrade Low Molecular Weight PVC

**DOI:** 10.1155/2016/9519527

**Published:** 2016-05-12

**Authors:** Tirupati Sumathi, Buddolla Viswanath, Akula Sri Lakshmi, D. V. R. SaiGopal

**Affiliations:** ^1^Department of Virology and DST-PURSE Centre, Sri Venkateswara University, Tirupati 517502, India; ^2^Department of Bionanotechnology, Gachon University, San 65, Bokjeong-dong, Sujeong-gu, Seongnam-si, Gyeonggi-do 461 701, Republic of Korea

## Abstract

One of the utmost man-made problems faced today has been the ever-increasing plastic waste filling the world. It accounts for an estimated 20–30% (by volume) of municipal solid waste in landfill sites worldwide. Research on plastic biodegradation has been steadily growing over the past four decades. Several fungi have been identified that produce enzymes capable of plastic degradation in various laboratory conditions. This paper presents a study that determined the ability of fungi to degrade low molecular weight polyvinyl chloride (PVC) by the enzyme laccase. We have isolated a fungal species,* Cochliobolus *sp., from plastic dumped soils and they were cultured on Czapek Dox Agar slants at 30°C. The effectiveness of this fungal species on the degradation of commercial low molecular weight polyvinyl chloride (PVC) was studied under laboratory conditions. Significant differences were observed from the FTIR, GC-MS, and SEM results in between control and* Cochliobolus *sp. treated PVC.

## 1. Introduction

Laccases (EC 1.10.3.2) are multicopper enzymes belonging to the group of blue oxidases which exist widely in nature and are defined in the Enzyme Commission (EC) nomenclature as oxidoreductases which oxidize diphenols and allied substances and use molecular oxygen as an electron acceptor [1, 2]. Laccases have been studied since 1883 when extracted first time from the* Japanese lacquer* tree and due to their ability to oxidize phenolic compounds [3–5]. However, the majority of laccases have been isolated from higher fungi like* Trametes *spp.,* Cerrena maxima*,* Coriolopsis polyzona*,* Lentinus tigrinus *and* Pleurotus eryngii*, and so forth, and additionally, laccases occur in saprophytic ascomycetes such as* Myceliophthora thermophila* and* Chaetomium thermophile *and are involved in the humification process of composts [6–13]. Therefore, the ability of laccases to oxidize a broad range of phenolic compounds employed in numerous industrial sectors has amplified their biotechnological potential.

The white-rot fungal organisms are used in biodegradation processes and the oxidative ability of these fungi is related to their extracellular and intracellular enzymatic system. Their nonspecific enzymatic system allows them to degrade a wide range of pollutants resistant to other microorganisms [14]. Two major steps have been developed for use of white-rot fungi for degradation of pollutants such as dyes, plastics, pesticides, chlorophenolic compounds, and nonphenolic compounds [15]. One is treatment with enzymes, whether purified or using broths from fungal cultures, and the second step is a direct degradation of pollutants using active cultures of fungi. The main advantage of fungi in the process of degradation lies in the broad range of enzymes produced and the further transformation of the intermediate biodegradation products. In addition, these laccases were used in easier operation and faster biodegradation of many xenobiotics [16]. The complex polymers are also degraded by the various fungi in the presence of LMS (Laccase Mediated System). Polyvinyl chloride (PVC), which was accidentally synthesized in 1872 by German chemist Baumann [17], has the molar mass of PVC which is 93.92596 ± 0.00090 g/mol and the pure PVC is soluble in tetrahydrofuran but insoluble in alcohol. PVC's relatively low cost, biological and chemical resistance, and workability have resulted in it being used for a wide variety of applications. However, the utilization of used PVC based plastics is still a serious problem and studies are in progress in finding proper methods that would lead to enhancing the biodegradation ability of such plastics. Actually the structure of PVC is similar to the structure of chlorophenolic compounds and the laccases ability to change their structural content. As per the best of our knowledge, this is the first report on production of laccase by* Cochliobolus* spp. and their ability in degrading PVC. Therefore, the main objective of this paper is to assess the ability of* Cochliobolus *sp. on the degradation of low molecular weight PVC with their laccase enzyme and the optimization of cultural and nutritional parameters for enhanced production of laccase.

## 2. Material and Methods

### 2.1. Collection of Soil Sample

Plastic dumped soil was collected from a plastic industry in Renigunta near to Tirupati, Chittoor district of Andhra Pradesh, India. The soil samples were collected from 5 inches below the soil surface and transferred to research laboratory in sterile polythene covers to isolate fungal cultures.

### 2.2. Isolation and Screening of Fungal Strain

The soil samples were sieved through 250 *μ*m pore size sieve and from each sample 1 g of soil sample was then added in different test tubes containing 10 mL physiological saline (NaCl, 8.5 g/L) and shaken well using vortex mixer. These test tubes were considered as stock cultures for different soil sample sites. From the stock cultures, a volume of 1 mL was transferred aseptically and added to a test tube containing 9 mL of sterile physiological saline and mixed well. From this test tube, 1 mL of aliquot was again transferred and mixed with another 9 mL of sterile physiological saline to make 10^−2^ dilution factor. Similarly, dilutions up to 10^−5^ were made using serial dilution technique for all soil samples. A volume of 1 mL of suspension from 10^−4^ and 10^−5^ serially diluted tubes was taken and spread evenly with sterile L-shaped glass rod over the surface of Czapek Dox Agar plates containing 0.02% guaiacol and incubated at 30°C 4 to 7 days, since laccase catalyzes the oxidative polymerization of guaiacol to form reddish brown zones in the medium [18].

### 2.3. Molecular Level Identification of Isolated Fungi

Based on the results of screening, one potential strain was selected for further studies. The potential isolate was observed for morphological characteristics and other SEM characters. The taxonomic identification of fungal species was done based on the ribosomal RNA and fungal systematic [19]. Finally the strain was identified by using 18s rRNA sequencing.

### 2.4. Medium and Culture Conditions for Laccase Production

To select the best medium for laccase production, five different media were screened and the five media used in the study were Czapek Dox medium, mineral salt medium, medium of Olga et al. (1998), medium of Coll et al. (1995), and basal salt medium and the pH values of all these media were adjusted pH 7.0 before autoclaving.

### 2.5. Effect of Cultural and Nutritional Parameters and Other Supplements on Laccase Production

The effect of pH on laccase production was studied by inoculating* Cochliobolus *sp. in 100 mL medium. The pH values of the media were adjusted to 5.0, 5.5, 6.0, 6.5, and 7.0 and all the flasks with growing culture of* Cochliobolus *sp. were withdrawn at different time intervals after inoculation for measurement of fungal biomass and extracellular protein content and activity of laccase in the culture filtrate. The influence of temperature on laccase production was studied by inoculating* Cochliobolus *sp. in 100 mL of medium. The inoculated media were incubated at different temperatures ranging from 20, 25, 30, 35, and 40°C and all the flasks with growing culture of* Cochliobolus *sp. were withdrawn at different time intervals for measurement of fungal biomass and extracellular protein content and activity of laccase in the culture filtrate. The effect of different carbon sources, namely, glucose, maltose, xylose, galactose, and lactose, on the production of laccase from* Cochliobolus *sp. was studied. The carbon sources were amended at the concentration of 1% in the medium. The pH of the medium was adjusted to 6.0 before sterilization. One mL of spore suspension of 7-day-old cultures of* Cochliobolus *sp. was transferred to 250 mL Erlenmeyer flaks containing 100 mL of medium amended with different carbon sources. All the flasks with growing culture of* Cochliobolus *sp. were withdrawn at different time intervals after inoculation for measurement of fungal biomass and extracellular protein content and activity of laccase in the culture filtrate. In order to find the suitable nitrogen sources for the maximum production of laccase from* Cochliobolus *sp. the nitrogen sources, namely, peptone, urea, ammonium nitrate, yeast extract, and ammonium sulphate, were amended at the concentration of 0.3% in the medium. All the flasks with growing culture of* Cochliobolus *sp. were withdrawn at different time intervals after inoculation for measurement of fungal biomass and extracellular protein content and activity of laccase in the culture filtrate.

### 2.6. Fungal Biomass Estimation

The cultures* Cochliobolus *sp. in the flasks were aseptically filtered through preweighed Whatman No. 1 filter paper to separate mycelial mat and culture filtrate. The filter paper along with mycelial mat was dried at 70°C in an oven until constant weight and this weight was recorded. Difference between the weight of the filter paper bearing mycelial mat and weight of only filter paper represented biomass of fungal mat. Fungal growth was expressed in terms of mg/flask.

### 2.7. Enzyme Assay

Laccase activity was measured at 30°C spectrophotometrically using guaiacol as a substrate with an absorbance coefficient value of 6800 M^−1 ^cm^−1^ at 470 nm [20]. The reaction mixture consisted of 10 mM guaiacol in 10% acetone (v/v) in sodium acetate buffer (100 mM, pH 5.0) and 1 mL culture filtrate. The mixture was incubated for 5 min and the absorbance was read at 470 nm. One unit (U) of laccase activity was defined as the amount of enzyme catalyzing the production of one micromole of coloured product per min per mL and expressed as number of Katals (1 mol of substrate conversion/s).

Calculation is as follows:

(1) Laccase activityU/mL=ΔA470/min×4×Vt×dilution factor€×Vs,

where *V*_*t*_ is final volume of reaction mixture. *V*_*s*_ is sample volume. € is extinction coefficient of guaiacol which is 6740 M^−1^ cm^−1^. 4 is derived from unit definition and principle. Protein was determined by following method of Lowry et al. [21] and the biomass was also estimated.

### 2.8. PVC Medium

Instead of carbon source, polyvinyl chloride (sigma-Aldrich) was used as a carbon source (3 g/100 mL) on agar plate for 3 months and with one-year time incubation in broth.

### 2.9. Statistical Analysis

All the experimental results presented in Tables 1 [Table tab2] [Table tab3] [Table tab4] [Table tab5] [Table tab6] [Table tab7] [Table tab8] [Table tab9] [Table tab10] [Table tab11] [Table tab12] [Table tab13] [Table tab14] [Table tab15] [Table tab16] [Table tab17]–18 are the mean of triplicate experimental results. Statistical comparisons using one-way analysis of variance (ANOVA) are followed by Duncan's test to find out significant difference between *P* ≤ 0.05 values of each sampling [22].

## 3. Results and Discussion

### 3.1. Isolation and Screening of Fungal Strain

**Figure 1:**
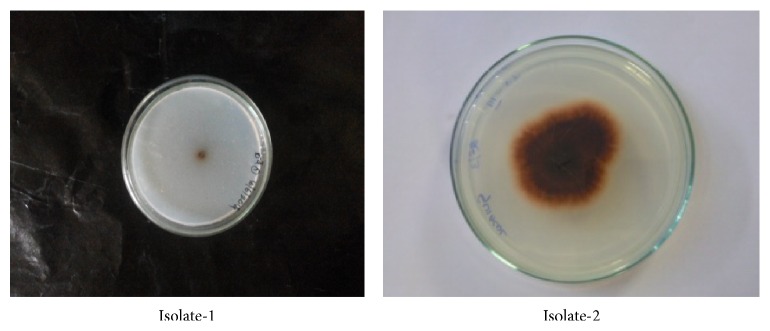
Isolated potential fungal strain showing oxidative polymerization of guaiacol to form reddish brown zones in the medium on 4th day.

Two morphologically different strains along with clear zones around the colony were observed in the Czapek Dox Agar medium after 4 days' incubation (Figure 1). The two strains were screened for production of laccase; both strains exhibited laccase activity on plate method. We have chosen one colony for further studies based on the enhanced laccase production during screening studies.

### 3.2. Morphological Test

**Figure 2:**
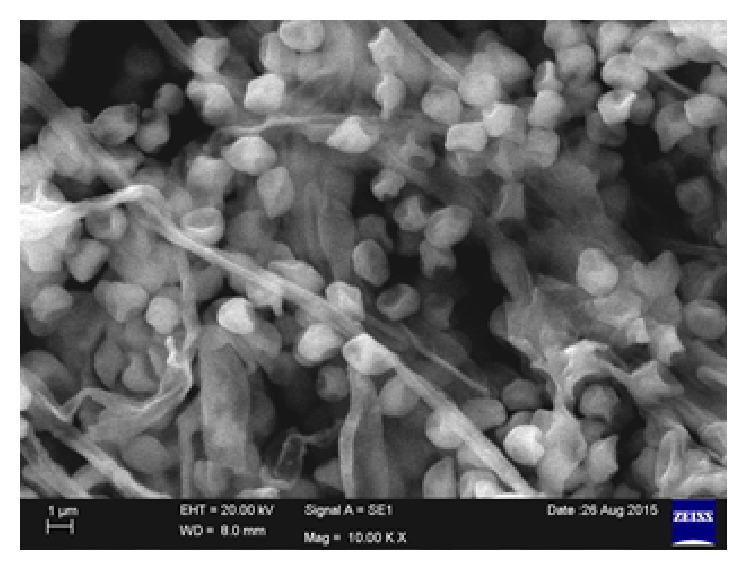
SEM image of potential fungal isolate-2.

When observed under SEM (Scanning Electron Microscopy, ZEISS Company, Sri Venkateswara University, Tirupati, Andhra Pradesh, India) we found the aerial mycelium monopodially branched spores bearing hyphae shape of fruiting bodies (Figure 2).

### 3.3. Identification of Fungal Strain

**Figure 3:**
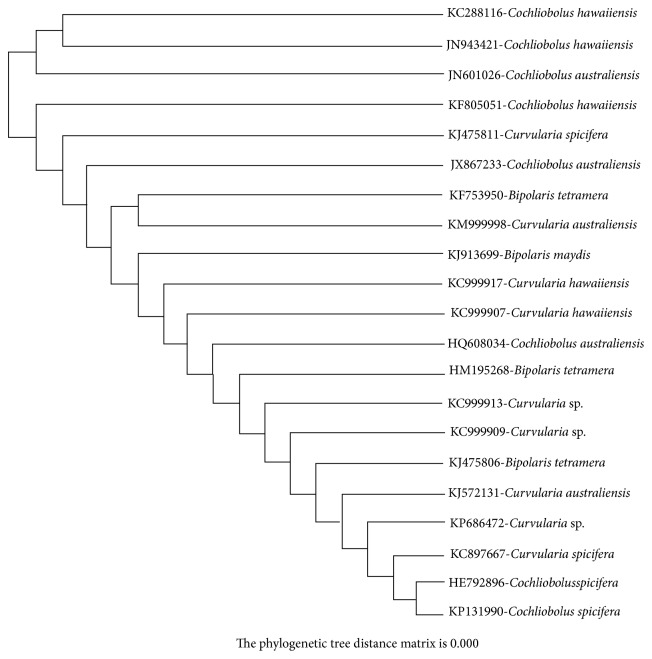
Phylogenetic tree of isolated potential fungi.

The taxonomic identification of potential fungal strain was based on 18s rRNA analysis. The 18s rRNA sequence of the strain was compared with the sequence in GenBank using BLAST and aligned with the sequence retrieved from NCBI GenBank databases using Clustal W method. The phylogenetic tree was constructed based on neighbour joining tree method illustrated in Figure 3. The database was deposited in NCBI GenBank with accession number KF805051. Based on the cultural, morphological, physiological, and molecular analysis the potential fungal strain was identified as* Cochliobolus *sp.

### 3.4. Medium and Culture Conditions for Laccase Production

To select the best medium for laccase production, five different media were screened and among the five media, Czapek Dox broth showed maximum laccase production (1.966 nKat/mL) and protein activity (2720 mg/mL) but maximum biomass (2.523 g/100 mL) was observed in Coll et al. medium on 6th day (Tables 1, 2, and 3).

### 3.5. Cultural and Nutritional Parameters and Other Supplements on Laccase Production

Physicochemical quantitative estimations were conducted at 20, 25, 30, 35, and 40°C. Production of fungal biomass, production of laccase, and total protein content varied with culture conditions. Maximum laccase production (1.793 nKat/mL) was recorded at 30°C on 6th day of incubation. In this case, maximum protein (2650 mg/mL) and the highest biomass (1.463 g/mL) were recorded and showed (Tables 4, 5, and 6) on the 6th day. Laccase production was minimum (0.526 nKat/mL) at 40°C with maximum production of protein content (0.426 mg/mL) and fungal biomass (310 mg/mL). While the production of laccase was recorded between pH 5 and 7, the maximum production of laccase (1.466 nKat/mL) along with protein content (2466.66 mg/mL) and biomass (1.450 g/100 mL) was favoured at pH 6.5 at 6th day of incubation (Tables 7, 8, and 9).

Among the five carbon sources xylose increased the production of laccase (1.743 nKat/mL), along with protein content (6690 mg/mL) and biomass (3.223 g/100 mL) (Tables 10, 11, and 12). Glucose is found as the second highest carbon source for the production of laccase, protein content, and biomass on 6th day of incubation. Other carbon sources (maltose, galactose, and lactose) were found to be less inhibitory for laccase production as well as fungal biomass. D'Souza-Tido et al. [23] screened different carbon sources for maximum laccase production by* Botryosphaeria *sp. They have screened glucose, fructose, galactose, galacturonic acid, xylose, lactose, sucrose, mannitol, pectin, and insulin and found increased production with most carbon sources studied except insulin and galacturonic acid. In this present study carbon source xylose produced maximum laccase enzyme. Among the five nitrogen sources, peptone was found to be the best source for the production of laccase (2.110 nKat/mL) and protein content (5926 mg/mL) (Tables 13, 14, and 15), but fungal biomass (3.223 g/100 mL) was the highest in ammonium nitrate. Similarly, in a study by Strong [24], peptone has improved laccase synthesis in Trametes pubescens MB89 to the greatest extent (1.8-fold increase) [24].

Addition of copper sulphate (CuSO_4_) increased laccase production at certain concentration. In recent study of white-rot fungus* Trametes trogii *[25] the addition of higher copper concentrations (500 *μ*M) inhibited the growth and laccase production. In another study by Palmieri et al. [26], the addition of copper sulphate (150 *μ*M) to cultural medium has increased the laccase production. In this present study 350 *μ*M/100 mL copper sulphate shows the highest production of laccase enzyme (Tables 16, 17, and 18) (0.904 nKat/mL) and protein content (950 mg/mL) and the highest in (300 *μ*M/100 mL) fungal biomass (0.790 mg/100 mL) but low compared with control.

### 3.6. PVC Medium

**Figure 4:**
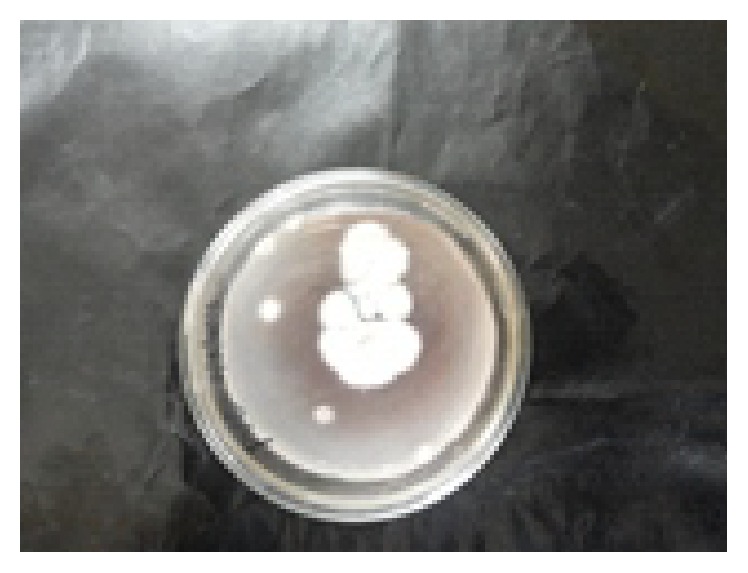
PVC as a carbon source utilized by potential fungal strain* Cochliobolus *sp.

**Figure 5:**
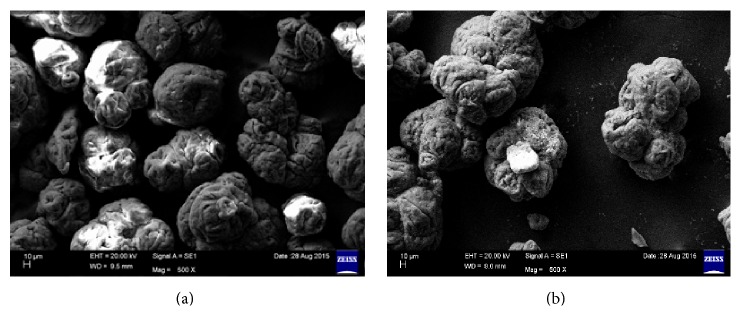
(a) PVC control SEM image. (b) PVC-BTS SEM image.

**Figure 6:**
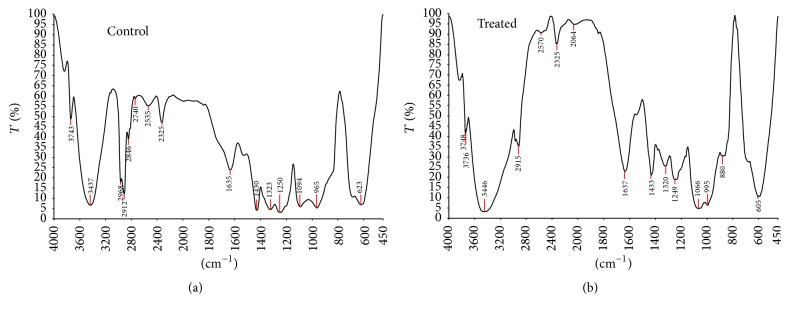
(a) FTIR results of PVC control sample. (b) PVC-biotreated sample, one-year incubation period.

**Figure 7:**
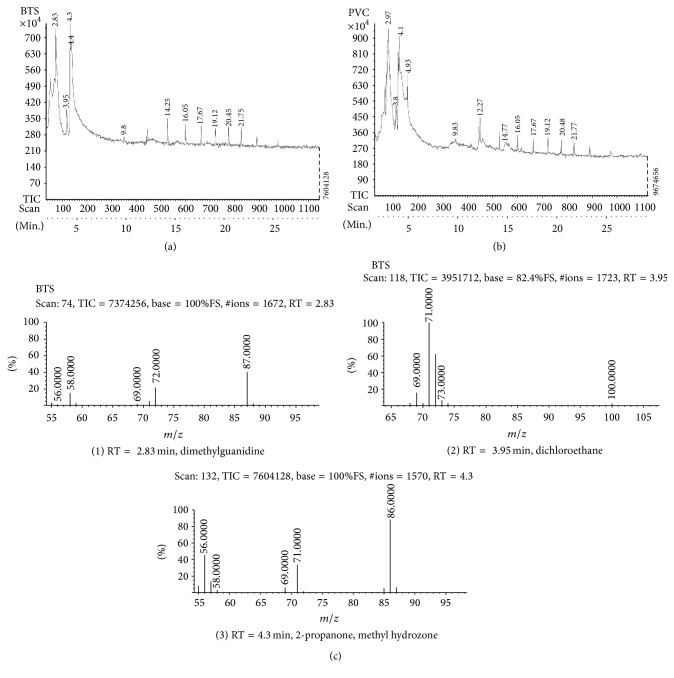
(a) Mass spectrum PVC-BTS treated with potential fungal strain chromatogram. (b) Mass spectrum of PVC control sample. (c) Chromatogram of substances with molecular weights.

In plate assay, the carbon source PVC was utilized by potential fungal strain as shown in Figure 4. In another study, the potential fungal strain was inoculated into optimized Czapek Dox broth amended with 3% PVC. The inoculated flask was incubated for one year on rotary shaker at 120 rpm (continuously) at room temperature. After incubation period broth was filtered and the PVC metabolites were subjected to distillation to remove fungal biomass. After distillation powder was collected and named as PVC-biotreated sample (BTS) and analysed through SEM and FTIR and the results were compared with control (PVC). The SEM results were showed at magnification of 500x in Figures 5(a) and 5(b) and FTIR and GC-MS analysis results were showed in Figures 6(a), 6(b), 7(a), 7(b), and 7(c) (1, 2, and 3). SEM results indicate the morphological and structural change in the treated PVC. So the fungal strains selected for the present study have the potential to degrade PVC. FTIR spectra of pure PVC showed CH-stretching mode at 2912 cm^−1^, -CH_2_ deformation mode at 1323 cm^−1^, C-H-rocking mode at 1250 cm^−1^, trans-C-H wagging at 965 cm^−1^, -C-Cl stretching mode at 810 cm^−1^, and cis-C-H wagging at 623 cm^−1^.

The characteristics of FTIR peaks of pure PVC (control) were found to have changed in the PVC-BTS (biotreated sample) system between 4000 cm^−1^ and 450 cm^−1^ wave number range. The peak at 2912 cm^−1^ was attributed to -C-H- stretching mode for pure PVC as shifting to 2915 cm^−1^ in the PVC-BTS. The appearance of new peaks at 2600 cm^−1^ to 2100 cm^−1^ and at 1100 cm^−1^ to 800 cm^−1^ in PVC-BTS, corresponding carbonyl group (C=O) confirmed the structural change in the treated PVC. The results show that some of the double bonds of PVC were cleaved by fungal strain. In addition, a decrease in the intensity and narrowing peaks can be observed in the PVC-BTS. These shifts are expected due to the interaction between PVC and BTS. Finally the FTIR results show that PVC was degraded by potential fungal strain and some structural changes also occurred. It indicates the biodegradation of PVC was done by the selected fungal strain. When compared with Sakhalkar and Mishra [27], GC-MS shows PVC-BTS which was pyrolyzed at 50°C with the composition of metabolites produced by microorganism (potential fungal strain) and the total chromatograms obtained at the same temperature; Figures 7(a) and 7(b) show the chromatogram of PVC-BTS sample treated with potential fungal sample and control (PVC). Based on the decomposition of products (Table 19), the biotreated sample was decomposed as different aromatic compounds like benzene; polycyclic aromatic hydrocarbons are also present in Table 19 and Figure 7(c). These indicate that fungal organism was degrading the PVC in one year time.

The decomposition of PVC treated with potential fungal strain indicates peaks 2-methyl-2-pentene (RT = 2.83 min), dichloroethane (RT = 3.95 min), 2-propanone, methyl hydrozone (RT = 4.3 min), 1,2-dichlorobenzene (RT = 9.8 min), 4-amino tetrahydropyran (RT = 14.25 min), 2,3-chlorobicyclo-oct-2-en-6-methyl-1,3-dioxolane (RT = 16.05 min), 1,2-benzenedicarboxylic acid (17.67 min), and so forth [28]. FTIR and GC-MS results from the present investigation indicate that PVC was degraded at significant level in this present study.

## 4. Conclusion

This investigation established the robust low molecular weight PVC degradation activity under lab conditions in which the synthetic polymer served as the only carbon source for the fungus,* Cochliobolus* sp. This work establishes that fungi are a useful source of biodiversity with potential application for bioremediation. In the present study, significant difference was observed from the FTIR, GC-MS, and SEM results in between control and* Cochliobolus *species treated low molecular weight PVC. Therefore, fungal treatment technology is effective and has potential to be used in full-scale remediation of plastic contaminated sites. The establishment of an economical and environment-friendly technique for PVC degradation using fungi, such as* Cochliobolus* sp., could be expected to provide a feasible solution to the plastic disposal problem.

## Figures and Tables

**Table 1:** Production of laccase (nkat/mL) by *Cochliobolus* sp. on different media.

S. number	Growth media	2nd day	4th day	6th day	8th day
(1)	Czapek Dox	0.806	1.660	1.966	0.406
(2)	Mineral salt	0.260	0.560	0.706	0.153
(3)	Olga et al.	0.640	1.240	1.346	0.306
(4)	Coll et al.	0.400	0.566	0.893	0.373
(5)	Basal salt	0.606	0.806	1.006	0.520

**Table 2 tab2:** Production of extracellular protein (mg/mL) by *Cochliobolus* sp. on different media.

S. number	Growth media	2nd day	4th day	6th day	8th day
(1)	Czapek Dox	973.33	1266.66	2720.00	573.33
(2)	Mineral salt	133.33	490.00	260.00	80.00
(3)	Olga et al.	526.66	583.33	933.33	166.66
(4)	Coll et al.	593.33	783.33	853.33	386.66
(5)	Basal salt	473.33	1073.33	1246.66	406.66

**Table 3 tab3:** Production of biomass (g/100 mL) by *Cochliobolus* sp. on different media.

S. number	Growth media	2nd day	4th day	6th day	8th day
(1)	Czapek Dox	0.236	0.623	1.406	0.526
(2)	Mineral salt	0.050	0.103	0.133	0.023
(3)	Olga et al.	0.586	1.573	2.190	0.853
(4)	Coll et al.	1.740	1.896	2.523	0.606
(5)	Basal salt	0.250	0.286	0.410	0.156

**Table 4 tab4:** Influence of initial pH of the media on laccase activity (nkat/mL) by *Cochliobolus* sp.

S. number	pH	2nd day	4th day	6th day	8th day
(1)	5	0.206	0.486	0.840	0.666
(2)	5.5	0.366	0.580	0.966	0.753
(3)	6	0.900	1.133	1.046	0.813
(4)	6.5	1.026	1.353	1.466	0.966
(5)	7	0.333	0.506	1.013	0.386

**Table 5 tab5:** Influence of initial pH of the media on extracellular protein (mg/mL) production by *Cochliobolus* sp.

S. number	pH	2nd day	4th day	6th day	8th day
(1)	5.0	426.66	1266.66	1640.00	540.00
(2)	5.5	576.66	910.00	2173.33	633.33
(3)	6.0	683.33	1620	2373.33	710.00
(4)	6.5	860	2060	2466.66	920.00
(5)	7.0	400	750	863.33	336.33

**Table 6 tab6:** Influence of initial pH of the media on biomass (g/100 mL) production by *Cochliobolus* sp.

S. number	pH	2nd day	4th day	6th day	8th day
(1)	5.0	0.126	0.216	0.280	0.173
(2)	5.5	0.273	0.543	0.813	0.783
(3)	6.0	0.313	0.623	1.096	0.983
(4)	6.5	0.433	0.753	1.450	1.133
(5)	7.0	0.246	0.283	0.643	0.180

**Table 7 tab7:** Influence of incubation temperature on laccase activity (nkat/mL) by *Cochliobolus* sp.

S. number	Temperature	2nd day	4th day	6th day	8th day
(1)	20°C	0.193	0.413	0.633	0.286
(2)	25°C	0.426	0.586	0.980	0.366
(3)	30°C	0.746	1.171	1.793	0.813
(4)	35°C	0.513	0.726	0.926	0.346
(5)	40°C	0.086	0.246	0.526	0.306

**Table 8 tab8:** Influence of incubation temperature on extracellular protein (mg/mL) production by *Cochliobolus* sp.

S. number	Temperature	2nd day	4th day	6th day	8th day
(1)	20°C	136.66	216.66	310	250
(2)	25°C	953.33	1086.66	1500	686.66
(3)	30°C	1720	1870	2650	1460
(4)	35°C	486.66	4460	533.33	293.33
(5)	40°C	106.66	193.33	310	243.33

**Table 9 tab9:** Influence of incubation temperature on biomass (g/100 mL) production by *Cochliobolus* sp.

S. number	Temperatures	2nd day	4th day	6th day	8th day
(1)	20°C	0.103	0.186	0.316	0.256
(2)	25°C	0.253	0.440	0.656	0.630
(3)	30°C	0.473	0.913	1.463	0.846
(4)	35°C	0.310	0.473	0.636	0.406
(5)	40°C	0.243	0.280	0.426	0.310

**Table 10 tab10:** Effect of different carbon sources on laccase activity (nkat/mL) by *Cochliobolus* sp.

S. number	Carbon sources	2nd day	4th day	6th day	8th day
(1)	Glucose	0.540	0.760	1.316	0.920
(2)	Maltose	0.380	0.540	1.046	0.820
(3)	Xylose	1.020	1.316	1.743	1.183
(4)	Galactose	0.386	0.453	0.680	0.480
(5)	Lactose	0.280	0.320	0.580	0.360

**Table 11 tab11:** Effect of different carbon sources on extracellular protein (mg/mL) production by *Cochliobolus* sp.

S. number	Carbon sources	2nd day	4th day	6th day	8th day
(1)	Glucose	2546.66	3220.00	5730.00	3416.66
(2)	Maltose	1346.66	2560.00	4350.00	2983.33
(3)	Xylose	3320.00	5060.00	6690.00	4433.33
(4)	Galactose	2626.66	4460.00	5183.33	3766.66
(5)	Lactose	986.66	2380.00	4140.00	2800.00

**Table 12 tab12:** Effect of different carbon sources on biomass (g/100 mL) production by *Cochliobolus* sp.

S. number	Carbon sources	2nd day	4th day	6th day	8th day
(1)	Glucose	0.233	0.440	2.450	1.863
(2)	Maltose	0.546	1.053	2.786	1.240
(3)	Xylose	0.643	1.830	3.223	2.510
(4)	Galactose	0.410	0.820	1.353	1.043
(5)	Lactose	0.310	0.743	1.270	1.010

**Table 13 tab13:** Effect of different nitrogen sources on laccase activity (nkat/mL) by *Cochliobolus* sp.

S. number	Nitrogen sources	2nd day	4th day	6th day	8th day
(1)	Peptone	0.533	1.743	2.110	1.153
(2)	Urea	0.173	0.493	1.356	0.786
(3)	Ammonium nitrate	0.226	0.740	1.506	0.966
(4)	Yeast extract	0.320	0.540	0.980	0.593
(5)	Ammonium sulphate	0.366	0.493	0.733	0.560

**Table 14 tab14:** Effect of different nitrogen sources on extracellular protein (mg/mL) production by *Cochliobolus* sp.

S. number	Nitrogen sources	2nd day	4th day	6th day	8th day
(1)	Peptone	2280.00	4066.66	5926.66	2586.66
(2)	Urea	746.66	1850.00	3196.66	1106.66
(3)	Ammonium nitrate	1946.66	2616.66	4806.66	2026.66
(4)	Yeast extract	733.33	2433.33	3593.33	1440.00
(5)	Ammonium sulphate	413.33	1883.33	2730.00	733.33

**Table 15 tab15:** Effect of different nitrogen sources on biomass (g/100 mL) production by *Cochliobolus* sp.

S. number	Nitrogen sources	2nd day	4th day	6th day	8th day
(1)	Peptone	0.220	0.850	1.156	0.243
(2)	Urea	0.220	0.726	0.966	0.326
(3)	Ammonium nitrate	0.550	1.863	3.223	0.486
(4)	Yeast extract	0.383	0.620	1.353	0.306
(5)	Ammonium sulphate	0.200	0.396	1.270	0.180

**Table 16 tab16:** Effect of CuSO_4_ concentration on laccase activity (nkat/mL) by *Cochliobolus* sp.

S. number	CuSO_4_ concentrations	2nd day	4th day	6th day	8th day
(1)	50	0.204	0.250	0.425	0.240
(2)	100	0.206	0.322	0.460	0.320
(3)	150	0.220	0.406	0.620	0.360
(4)	200	0.260	0.463	0.710	0.420
(5)	250	0.220	0.640	0.802	0.540
(6)	300	0.240	0.802	0.840	0.700
(7)	350	0.250	0.606	0.940	0.806
(8)	400	0.232	0.506	0.740	0.606
(9)	450	0.280	0.360	0.620	0.420
(10)	Control	0.646	1.940	2.432	1.298

**Table 17 tab17:** Effect of CuSO_4_ concentration on extra cellular protein (mg/mL) production by *Cochliobolus* sp.

S. number	CUSO_4_ concentrations	2nd day	4th day	6th day	8th day
(1)	50	243	353	553	353
(2)	100	243	406	563	380
(3)	150	326	406	623	446
(4)	200	366	563	723	553
(5)	250	326	634	806	653
(6)	300	340	834	846	753
(7)	350	450	686	940	853
(8)	400	432	516	756	646
(9)	450	280	360	680	480
(10)	Control	646	1940	2432	1298

**Table 18:** Effect of CuSO_4_ concentration on fungal biomass (g/100 mL) production by *Cochliobolus* sp.

S. number	CuSO_4_ concentrations	2nd day	4th day	6th day	8th day
(1)	50	0.250	0.286	0.410	0.156
(2)	100	0.340	0.446	0.566	0.260
(3)	150	0.246	0.546	0.633	0.336
(4)	200	0.240	0.356	0.723	0.453
(5)	250	0.150	0.470	0.943	0.643
(6)	300	0.236	0.616	1.406	0.790
(7)	350	0.216	0.560	0.653	0.330
(8)	400	0.363	0.480	0.586	0.586
(9)	450	0.466	0.660	0.740	0.353
(10)	Control	0.556	0.773	2.632	1.410

**Table 19:** Mass spectrum of substances PVC treated with *Cochliobolus* sp. (BTS) with RT = retention time of substance chromatogram.

Peak	Retention time (RT) (min)	Pyrolysis BTS treated with PVC at 500°C	Substance molecular weight (g/mol)
(1)	2.83	Dimethylguanidine	87
(2)	3.95	Dichloroethane	100
(3)	4.3	2-Propanone, methyl hydrozone	86
(4)	9.8	1,2-Dichlorobenzene	147
(5)	14.25	4-Amino tetrahydropyran	150
(6)	16.05	2,3-Chlorobicyclo-oct-2-en-6-methyl-1,3-dioxolane	198
(7)	17.67	1,2-Benzenedicarboxylic acid	222
(8)	19.12	5.5-Bipthalide	266
(9)	20.45	5-Chloro-(2,4-dichlorophenoxy) phenol	280
(10)	21.75	Diethyl [(phenyl sulfonyl)methyl] phosphonate	295
